# SugarViT—Multi-objective regression of UAV images with Vision Transformers and Deep Label Distribution Learning demonstrated on disease severity prediction in sugar beet

**DOI:** 10.1371/journal.pone.0318097

**Published:** 2025-02-13

**Authors:** Maurice Günder, Facundo Ramón Ispizua Yamati, Abel Barreto, Anne-Katrin Mahlein, Rafet Sifa, Christian Bauckhage

**Affiliations:** 1 Fraunhofer Institute for Intelligent Analysis and Information Systems IAIS, Schloss Birlinghoven, Sankt Augustin, Germany; 2 Institute for Computer Science III, University of Bonn, Bonn, Germany; 3 Institute for Sugar Beet Research (IfZ), Göttingen, Germany; King Fahd University of Petroleum & Minerals, SAUDI ARABIA

## Abstract

Remote sensing and artificial intelligence are pivotal technologies of precision agriculture nowadays. The efficient retrieval of large-scale field imagery combined with machine learning techniques shows success in various tasks like phenotyping, weeding, cropping, and disease control. This work will introduce a machine learning framework for automatized large-scale plant-specific trait annotation for the use case of disease severity scoring for CLS in sugar beet. With concepts of DLDL, special loss functions, and a tailored model architecture, we develop an efficient Vision Transformer based model for disease severity scoring called SugarViT. One novelty in this work is the combination of remote sensing data with environmental parameters of the experimental sites for disease severity prediction. Although the model is evaluated on this special use case, it is held as generic as possible to also be applicable to various image-based classification and regression tasks. With our framework, it is even possible to learn models on multi-objective problems, as we show by a pretraining on environmental metadata. Furthermore, we perform several comparison experiments with state-of-the-art methods and models to constitute our modeling and preprocessing choices.

## Introduction

In precision agriculture, the use of UAV equipped with multispectral cameras for monitoring agricultural fields is well-established for various tasks regarding plant phenotyping and health status [1–6]. Especially in phenotyping for breeding, one of the main advantages of UAV imagery is the mapping flexibility in comparison to satellite imagery, automation and homogenization of laborious and time-consuming visual scoring activities, which usually require large numbers of hours of specialized human labor to score large fields. In agriculture, the term “visual scoring” is commonly used for a field assessment, such as phenotyping canopy structure or the quantification of disease intensity, specifically, DS [7]. On the other hand, in data science, a related procedure called “annotation” is used. Annotation consists of labeling data elements in order to add semantic information or metadata. In essence, although the terms differ in their apparent applications, they represent in this paper an equivalent concept, and in this context, we will use “annotation” as a synonym for “scoring”. Theoretically, when large field experiments are conducted, this data is immediately available. However, a lot of human-powered effort is needed to gain information out of large imagery. This is where machine learning comes into play. (Rehashed) UAV image data has the potential to serve as training data for even large image-processing deep learning models. In recent years, the visual disease severity assessment with mainly CNN based models has made a significant progress [8]. With breakthrough results regarding the application of transformer-based model architectures in diverse research areas [9], they are increasingly used for disease severity assessment as well. The origin of the transformer architecture lies in the field of language processing and has led to large success in recent large language models such as the GPT [10]. The basic principle of transformers is the so-called attention mechanism [11]. It enables the model to connect and associate features over large semantic or sequential distances. This is beneficial not only for one-dimensional tasks as language processing, since this context can be transferred to higher dimensional use cases like image processing. In this case, we are dealing with a ViT [12] model. Recent works like [13] and [14] use ViT for plant disease localization and classification.

With the power of transformers also a major drawback appears, namely their low data efficiency. Transformers need lots of data to train. This is why their success is currently mainly in application fields where large datasets are available, such as text data. However, we will show, that also on large-scale agricultural datasets enabled by UAV, those models can be used for annotation tasks. To demonstrate this potential in our work, we will focus on a classification task based on single plant images extracted from recorded sugar beet fields according to Günder et al. [15]. The single sugar beet plant images are annotated with DS estimations of CLS, a fungal leaf disease that is a relevant disease causing yield losses in sugar beet production [26]. We aim a DS prediction modeling task and will motivate a multi-objective approach as well as the use of a deep learning architecture based on a ViT. In contrast to [13] and [14], we identify the DS prediction as an ordinal classification and reinterpret the classification as a regression task by using the concept of DLDL introduced in [17]. We further optimize the vanilla DLDL approach by an improved loss function that does not need a proper hyperparameter tuning [18]. After the training, our model, we further call SugarViT, is able to predict the disease severity of individual plant images by a probability distribution which gains training robustness and output interpretability. With its ViT backbone, SugarViT is able to estimate the DS more accurately than with convolution-based backbones of comparable complexity. All in all, SugarViT demonstrates a novel, robust, and flexible approach to automatized disease severity annotation for precision agriculture; based on UAV-imagery and aided by environmental data.

In the following work, we will go into all the details of SugarViT and the conducted experiments starting from the methodology and foundational concepts.

## Materials and methods

In this section, we shed light on the underlying data for our model and the associated preprocessing methods. Thereafter, we focus on the use case and the model properties consisting of architecture and the objectives.

### Data and preprocessing

A major challenge that comes with the application and, particularly, the training of vision transformer based architectures requires large amounts of image data. In principle, the utilization of UAV imaging from crop fields has the potential to gain those large datasets. However, the conditions under which the images are taken can be very diverse, e.g., due to variable weather, lighting, and resolution. Additionally, device-specific properties can come into play when dealing with different camera models or calibration methods. In the context of plant phenotyping, it is particularly desirable to accumulate image data from multiple growing seasons, which implies that all the above-mentioned difficulties can play a role for an accumulation of large-scale datasets. Thus, in order to utilize as much potential from the data, a preprocessing is needed, that is robust against as many confounding factors as possible.

#### Available field data.

The dataset we use in this work consists of multispectral images expressed in reflectance of single sugar beet plants recorded by UAV systems on 6 different locations near Göttingen, Germany (51 ∘ 33′N 9 ∘ 53′E) [15]. UAV systems are equipped with a multispectral camera recording 5 spectral channels. Those are, sorted by wavelength, blue, green, red channel, REDGE, and NIR. Due to a large amount of different sensors, sugar beet varieties, resolutions (or ground sampling distances), locations, and time points, the dataset is very diverse. In total, it covers 4 harvesting periods from 2019 to 2022 and comprises 17 different experiments or flight missions. Table 1 gives an overview over all important information of the dataset. Additionally, Table 2 shows the spectral bandwidths of the two camera sensor systems used in this work.

**Table 1:** Overview of datasets. All images are given as 5-channel multispectral data with 144*px* × 144*px* of size. The abbreviation GSD stands for ground sampling distance.

ID	sowing date	location	sensor	GSD mm px^-1^	varieties	recordings	images	used for
Tr01	2019-04-09	Holtensen I	RedEdge	3.5	2	23	13428	train/val
Tr02	2019-04-09	Holtensen I	RedEdge	15	2	21	18157	train/val
Tr03	2020-04-06	Weende	ALTUM	3	2	25	37905	train/val
Tr04	2020-04-06	Weende	ALTUM	4	1	25	240120	train/val
Tr05	2021-04-01	Sieboldshausen	ALTUM	5.1	51	7	39527	train/val
Tr06	2021-04-23	Holtensen I	ALTUM	3	5	27	112655	train/val
Tr07	2021-04-23	Holtensen I	ALTUM	4	1	23	212559	train/val
Tr08	2021-04-23	Holtensen I	ALTUM	3	1	22	35878	train/val
Te01	2021-04-23	Holtensen I	ALTUM	5	1	22	45701	test
Tr09	2021-04-30	Dramfeld	ALTUM	4	1	22	33484	train/val
Tr10	2021-04-30	Dramfeld	ALTUM	5	1	22	30530	train/val
Tr11	2022-04-19	Weende	ALTUM	4	1	12	203139	train/val
Tr12	2022-04-20	Reinshof	ALTUM	5	4	11	21301	train/val
Tr13	2022-04-20	Weende	ALTUM	5.1	4	12	15604	train/val
Tr14	2022-03-31	Reinshof	ALTUM	9	1	11	11406	train/val
Tr15	2022-03-31	Reinshof	ALTUM	18.5	1	8	7065	train/val
Tr16	2022-03-31	Holtensen II	ALTUM	3.4	1	21	59639	train/val
						total	1092397	train/val
							45701	test

**Table 2:** Spectral ranges of the two camera sensor systems used in this work. The thermal band is not used in this work.

band name		sensor	
long	short	ALTUM	RedEdge
blue	B	459 - 491*nm*	465 - 485*nm*
green	G	548 - 572 *nm*	550 - 570 *nm*
red	R	661 - 675*nm*	663 - 673*nm*
red edge	REDGE	711 - 723 *nm*	712 - 722 *nm*
near infrared	NIR	814 - 870*nm*	820 - 860*nm*
thermal	TH	8 - 14 μ*m*	-

All experiment fields are equipped with weather sensors, allowing for hourly temperature and humidity measurements in the fields. We can use this data to infer some more environmental quantities. We particularly focus on two of them. Firstly, a basic yet widely used quantity in phytology that connects the local weather with the development stage of the crop is the cumulative GDD. For each day, the plant accumulates a so-called *thermal sum* calculated by


$$\begin{array}{ll} GDD=\frac{1\;d}{24}\overset{23}{\underset{t=0}{\sum }}\left(\frac{T_{\text{max}}(t)+T_{\text{min}}(t)}{2}-T_{\text{base}}\right). & \; \end{array}$$ (1)

For the hourly maximum and minimum temperatures $T_{\text{max}}(t)$ and $T_{\text{min}}(t)$ additionally applies an upper and lower bound


$$\begin{array}{l} T(t)=\{\begin{array}{cc} T_{\text{max}},\; & \;T(t)>T_{\text{max}}\\ T_{\text{base}},\; & \;T(t)<T_{\text{base}}\\ T(t)\; & \text{else} \end{array}\;, \end{array}$$ (2)

where the base and maximum temperatures $T_{\text{base}}$ and $T_{\text{max}}$ are plant-specific parameters. For sugar beet, it is empirically shown that $T_{\text{base}}=1.1\;\circ C$ and $T_{\text{max}}=30\;\circ C$. [19]. The cumulative quantity of GDD beginning at the sowing date is, after all, a proxy of the plant’s development. Secondly, we can calculate a disease-specific quantity. Simply put, the time between the infection of a plant with Cercospora and the ability of infecting other plants is called a *generation* or *incubation period*. The thermal sum of one incubation period for Cercospora in sugar beet is found to be 4963°*C* × *h* with $T_{\text{base}}=6.3\;\circ C$ and $T_{\text{max}}=32\;\circ C$ [20]. For each hourly summand, there is an additional empirical correction coefficient based on the relative humidity: the hourly summand is multiplied by $\frac{7}{8}$ if the hourly relative humidity is less than 80*%*. If it is at least 80*%*, the summand is multiplied by $\frac{9}{8}$. [21,22] Summation of the thermal sum and division by the incubation period yields a quotient, that describes the potential number of incubation periods a hypothetically infected plant could have undergone. We call this the NPG. Thus, given environmental information, we can calculate field- and recording-date-specific parameters that can serve as additional data to support the individual plant image data.

#### Image normalization.

In the vast majority of machine learning tasks dealing with image processing, images are normalized to ensure interoperability and robustness against varying image recording conditions. Additionally, numerical issues in forward and backward pass to deep learning architectures lead to the usage of data values around zero. A naive yet common approach is a simple standardization of the image data by subtracting the channel-specific mean *μ_c_* or a global mean *μ* and division by the std $\sigma_{c}$ or *σ*, respectively, for each image channel *C* in the image *I*. The standardization can be done with precalculated (channel-wise) means and std by using the information of the whole given dataset, with image specific means and std, or even with fixed, suggested values. In this work, we will assume that our reflectance images dataset could possibly have a bias. Therefore, we standardize each image by only using its own information. Further, we differ between channel-wise and a total standardization by using means and std for each channel separately and cross-channel calculations, respectively. Thus, we get channel-wise standardized images $I_{s}^{\text{ch}}$ and total standardized images $I_{s}^{\text{tot}}$ by


$$\begin{array}{lll} I_{s}^{\text{ch}} & ={\left\{\frac{C-\mu_{c}}{\sigma_{c}}\right\}}_{C\in I}\;,\; & \text{(3)}\;\\ I_{s}^{\text{tot}} & =\frac{I-\mu }{\sigma }\;.\; & \text{(4)}\; \end{array}$$

**Fig 1:**
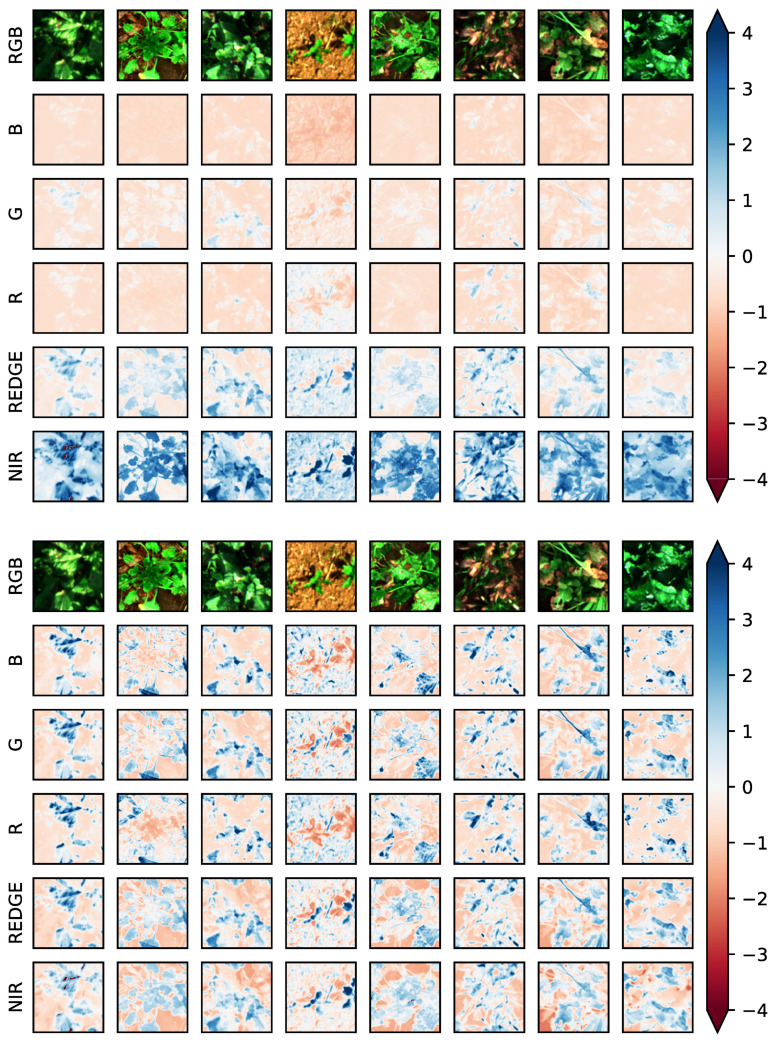
Example images shown by its separate channel components and processed with total (top image grid) and channel-wise standardization (bottom image grid), respectively. The first row in both grids shows the RGB representation.

Fig 1 shows some example images separated into its channel components and normalized with those two standardization methods. It is clearly visible, that the total standardization preserves the reflectance differences of the channels due to the emission spectrum of the plants and results in very distinct image channels. Thus, this standardization method emphasizes spectral characteristics of the image. In contrast, the channel-wise standardization results in rather similar image channels emphasizing local characteristics of the image in each channel.

A more sophisticated normalization method that, however, comes with more computational effort, makes use of the image histogram, i.e. the abundance of data values in the images. The HE is a contrast enhancement method that is broadly used in computer vision and image processing task for many application fields like medical imaging, as well as for signal processing like in speech recognition [23]. Briefly spoken, with respect to images, particularly, the idea is to normalize the elementary pixel values by their abundance. Thus, the number of pixels in each bin, or range of contrast, is equalized. As a result, each image is forced to use the full range of possible contrast.

Generally, there are two basic methods—local and global. Unlike global methods, local methods additionally use the environment of the corresponding pixel for equalization. They are usually grouped under the term *Adaptive Histogram Equalization* (AHE) where a prominent method is called *Contrast Limited AHE* (CLAHE) [24]. The adaptive methods are used for image processing tasks, where the pure contrast between neighboring objects is important, like in medical applications for tomography images [25]. In this work, we apply the HE method and introduce a channel-wise and cross-channel variant analogous to the standardization. For the histogram-based methods, a lower and upper limit has to be predefined to which the values are scaled. In this work, we chose the values to be in the range  [ - 1 , 1 ] .

#### Data augmentation.

A common approach to artificially increase the dataset size is data augmentation. For image data, the principle is to sample similar images around “real” data instances by various techniques like flip, rotation, color and brightness jitter, etc. Obviously, different use cases allow for different augmentation methods. For instance, in case of medical imaging task, one is mostly bound to the image orientation. In case of street scene images for autonomous driving applications, a vertical flip, i.e. put the image upside down, does not make any sense. However, both cases eventually allow for changes in brightness and/or contrast. In our case of plant images, we fortunately have all degrees of freedom regarding flips and rotations. Thus, we flip each image randomly with a probability of 25*%* and rotate each image by a random angle. Brightness and contrast jitters are not necessary, since our normalization methods neutralize them. Additionally, we can exploit this principle in model inference mode by evaluation the images in different rotations and average the predictions. In order to be robust against different ground sampling distances and accompanied resolution changes, we introduce a Gaussian blur augmentation. With a probability of 10*%*, an image is blurred at a strength of 3 - 8*px*. Another optional augmentation, is a random channel dropout. With a probability of 25*%*, we drop the information of up to 3 channels. Although it is quite unlikely, that in the application, single channels will be dropped, it is interesting to train models being robust against missing information in order to see, how important each image channel is for the prediction of our target quantity. With the channel dropout, we lower the ability of the model to focus on single channels and rather connect information among all channels.

Next, we shed light on one purpose the data has been recorded for and how we will use it for the use case described in this work.

#### Use case: disease severity estimation.

We will focus on the disease CLS, one of the most damaging foliar diseases in sugar beet cultivation. It is caused by the fungus *Cercospora beticola*. Symptoms appear as numerous small, round, gray spots with a red or brown border on leaves. As the infestation increases, the leaves become necrotic and dry up. When a large part of the leaf area is lost, the plant often tries to recover by generating a new birth of leaves at the cost of its stored sugar. However, if the conditions are favorable for the fungus and the attack is very severe, the plants die. [26,27] In this work, the goal behind our use case is to determine the DS in CLS-infected sugar beet fields. Generally, the observation and assessment of plant diseases is usually done by visual scoring. Being the visual field scoring of DS an activity that requires a lot of time and well-trained personnel [28], it is the main bottleneck in the control of CLS. Therefore, it is desirable to have an automatic DS estimation model.

Considering heterogeneous disease distribution within sugar beet fields, a detailed and geo-referenced assessment of DS might lead to precise protection measures within the canopy. Geo-referenced and plant-based determination of DS is therefore essential. A naive approach for the prediction of DS with single plant images would be to model a classification problem like in [29]. Despite being a valid approach at first sight, certain phenotypical knowledge enables to model this problem more intelligently. In the following sections, we will explain our basic paradigm to solve the DS estimation problem and our proposed deep learning model based on it.

**Fig 2:**
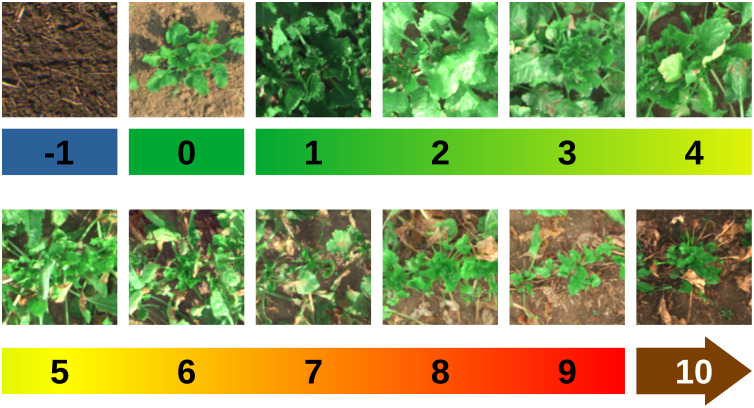
Used disease severity scale for our prediction model with example images. The scale is based on the usual CLS rating scale complemented with our definitions for  - 1, 0 and 10.

First, we have to define a DS annotation scale that serves as a guideline for all human expert annotations and finally as “unit” of the model input. In this work, the rating scale developed in [30] will be used with an extension for non-infested and newly sprouted plants. Fig 2 shows the numerical scale with exemplary plant images. The scale from 1 to 9 belongs to definitions of the KWS scale. The KWS scale is a severity diagram that ranges from 1 to 9. A rating of 1 indicates the complete absence of symptoms, while a rating of 3 indicates the presence of leaf spots on older leaves. A rating of 5 signifies the merging of leaf spots, resulting in the formation of necrotic areas. A rating of 7 is assigned when the disease advances from the oldest leaves to the inner leaves, leading to their death. Finally, a rating of 9 is given when the foliage experiences complete death [31]. In order to complement the scale, we added the 0 for non-infested sugar beets before canopy closure, and the 10 for newly sprouted plants as in [30]. In order to apply the model also on regions, where only soil is visible, we further added a  - 1 as “no plant” or “soil only” label by still maintaining the continuous fashion of the severity scale. This should also increase the model’s focus on the plant because it learns examples where no plant is visible at all.

**Fig 3:**
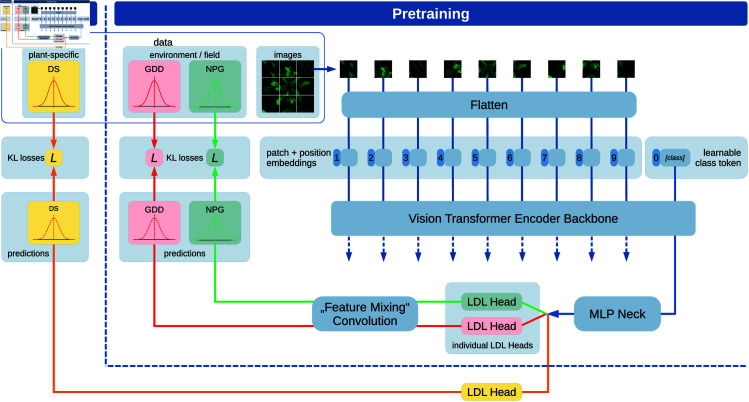
Sketch of our proposed Multi Deep Label Distribution Learning (Multi-DLDL) network with a ViT backbone. The LDL heads are trained with separate optimizers and loss functions. The ViT and MLP part are the joint basis and are trained in each backward pass of the LDL heads. As output of the ViT, the last hidden state of the learnable class token is used. Furthermore, our use case is shown by having multispectral plant image data and two training stages. The pretraining is done on the environmental, field-related quantities GDD and NPG. The target label DS is trained in the subsequent finetuning stage. In principle, the model can be generalized to more labels in each training stage by adding more LDL heads.

In field experiments, we often face data or annotations that need a high effort to acquire. Nevertheless, several data can be acquired rather automatically or with low human effort. In this work, we call this “cheap” and “expensive” labels. The DS acquisition is rather expensive, while, for instance, weather data acquired with automatic sensors or public weather stations is, typically, relatively cheap. Additionally, the development and epidemiology of the pathogen and disease CLS is highly influenced by specific environmental conditions. [32] In this work, we will make use of the cheap data in order to increase efficiency on expensive data. As shown above in Section *Available field data*, we have the weather-based parameters GDD and NPG. They are not plant specific but, at least, specific for the recording date. Thus, we can annotate many plants with those labels at one stroke. Those labels are, surely, not as meaningful as manually annotated labels, but they can serve for pretraining models. This is particularly interesting for our application of transformers, since they usually need lots of data: we can pretrain the model with the cheap labels and finetune on the expensive labels. Thus, a possible lower availability of the expensive labels could be compensated and training speed is enhanced if the model backbone at the start of finetuning stage already “knows” low-level filters and the basic concept of our input data. The two different stages of pretraining and finetuning are represented as different learning paths in our model sketch in Fig 3. Additional details of the model are discussed in the later sections of this work. First, we want to introduce in the concept behind our model architecture and, secondly, we shed light on the different model parts in detail.

### Deep Label Distribution Learning

If classification problems can be formulated within an ordinal scale, the transfer into a regression task might be a good choice. However, if the classification is very granular, the collection of data with precise labels can be challenging. Rather than learning distinct, unique labels, the paradigm of LDL [33] was proposed. It stabilizes the model training of labels by modeling their ambiguity. It is used for tasks like facial age estimation [34] or head pose estimation [35]. In combination with deep neural networks, the paradigm is referred to as DLDL [17]. In DLDL, the output of a deep neural network mimics the label distribution by a series of neurons that learn a discrete representation of the probability density function. This representation is commonly known as the pmf. Thus, the labels have the form of a probability distribution and the obvious difference in contrast to a pure regression is that the network output is not only based on a single neuron, whereas the difference to a pure classification is that, in contrast to one- or multi-hot-labels, also neighboring neurons are triggered which stabilizes regions, where fewer data is available. Two additional advantages, especially for the use case in this work, are, firstly, that we can easily model uncertainty of labels. The DS annotation is based on individual human experts’ judgement. Often, different plants are annotated by different experts, which causes uncertain classifications. Secondly, the model output becomes more transparent, since one can observe how confident the model is in its prediction by comparing the shapes of true and predicted label distribution. Thus, DLDL, once more, is an ideal way to model these annotations.

#### Full Kullback-Leibler divergence loss.

The DLDL approach proposed in [17] utilizes a L1 loss for the expectation value and a KLD [36] loss for the label distribution. However, L1 and KLD loss originate from different statistical concepts and, therefore, have scales that are, per se, not comparable. In most cases, a weighting parameter has to be introduced, resulting in an artificial hyperparameter of the model. Our approach circumvents the problem by reformulating the L1 loss as a KLD loss. Additionally, we further accelerate the training by introducing a “smoothness” regularization to the label distribution. The regularization is also formulated as a KLD loss, not needing any hyperparameter, either. Furthermore, the gained scale invariance not only makes the components comparable, but also enables the cross-comparability between different labels. This is especially interesting in the use case of this work, since we aim a joint regression of diverse phenological parameters, probably having different domains. This novel loss function is already introduced in [18]. However, since the approach is very well suited for the use case in this work, we will introduce the 3 loss components again in the following.

*Label distribution loss.* Let *ℙ* ( *y* ∣ *x* )  be the true label distribution for a given data point, i.e. an image, *x*. Then, the label distribution loss *L_ld_* is the discrete Kullback-Leibler divergence between the true and predicted label distribution,


Lld=KLD⁡ (ℙ||ℙ^)= ∑yℙ(y∣x)log ⁡ ℙ(y∣x)ℙ^(y∣x), (5)

where the hat denotes the prediction. This definition follows the label distribution loss given in [17].

*Expectation value loss.* Unlike in [17], we formulate the expectation value loss as a KLD of truth and prediction as if both of them were normal distributions $N(\cdot |\mu ,\sigma^{2})$ with expectation value *μ* and variance σ^2^. For the model predictions, $\hat{\mu }$ and ${\hat{\sigma }}^{2}$ can be calculated via


$$\begin{array}{ll} \hat{\mu }=\text{E}[\hat{\mathbb{P}}]=\underset{y}{\sum }y\hat{\mathbb{P}}(y|x)\;,\;{\hat{\sigma }}^{2}=\text{Var}[\hat{\mathbb{P}}]=\underset{y}{\sum }{\left(y-\hat{\mu }\right)}^{2}\hat{\mathbb{P}}(y|x)\;. & \; \end{array}$$ (6)

Thus, our expectation value loss is


Lexp=KLD ⁡ (N(⋅∣μ,σ2)||N(⋅∣μ^,σ^2))=log ⁡ σ^σ-12+σ2+(μ^-μ)22σ^2. (7)

Detailed calculation steps can be found in the Appendix of [18].

*Smoothness regularization loss.* In order to accelerate the training process, especially in early stages, we force the predicted label distribution to be smooth by a KLD regularization term. The idea is to shift the predicted distribution by one position which we call ${\hat{\mathbb{P}}}^{s}$ and calculate a symmetric discrete KLD, i.e. we average both shift directions. Thus,


Lsmooth=12 [KLD ⁡ (ℙ^||ℙ^s)+KLD ⁡ (ℙ^s||ℙ^)]=12∑y(ℙ^(y∣x)-ℙ^s(y∣x))log ⁡ ℙ^(y∣x)ℙ^s(y∣x). (8)

Finally, a sum combines the loss components. Thus, our final loss is


$$\begin{array}{ll} L=L_{ld}+L_{exp}+L_{smooth}. & \; \end{array}$$ (9)

#### Multi-head regression.

If multiple sources of labels are available, it may be considerable to perform the regression with multiple labels jointly. Each regression problem is then realized by an own so-called “regression head”, i.e., a sub-model, that is trained to transform the feature representation from the backbone into the respective label space of interest. Especially for large backbone models, this has the advantage that only one backbone is needed for multiple purposes, which reduces the total model size. We further call this concept “Multi-Head Regression”.

### Model architecture

In this chapter, we shed light on the architecture of our proposed model. Fig 3 shows all the building blocks of our proposed model, further called “SugarViT”. We now describe the 3 main building blocks of SugarViT and its design motivations.

#### Vision transformer backbone.

In recent years, transformer architectures are successfully utilized for diverse deep learning tasks. The underlying attention mechanism [11] is able to relate patterns and semantics in sequential data very flexible and across large structural distances. Especially in the field of NLP, transformer-based models show great success [10]. In NLP, transformers learn structures in sequential data like text or sentences by processing its basic building blocks, commonly knows as “tokens”. To use this principle also for classification tasks on image data, the Vision Transformer (ViT) model has been proposed. [12] The main principle is to divide an input image into flattened tiles that are processed by several multi-head attention layers. An additional learnable “class token” is added, which processed output is passed through a classification head. Fig 3 also shows the mentioned building blocks. By comprising many attention blocks and hidden layers, (vision) transformer architectures are complex and need large amounts of data. Thus, they are usually pretrained on large-scale datasets like ImageNet [37] for most of the image processing tasks. For the use case this work is about, we process multispectral 5-channel images rather than RGB images. Therefore, we cannot use ImageNet-pretrained architectures per se. However, the plant image dataset used in this work is large enough to train an architecture with a vision transformer backbone from scratch, as we will see in further sections. The goal of the learning process is that the ViT backbone is trained to be an expert in understanding the image as a whole and extract remarkable traits to “encode” the image information into a rich feature space.

#### MLP neck.

In the original ViT model, an MLP is used as a classification head. Since we want to build a multi-head regression model (cf. Section *Multi-head regression*), we use an MLP as an intermediate layer between the ViT backbone and the regression heads. If certain labels have something in common or share some information, i.e., latent label correlations, this “neck” sub-model between backbone and heads is trained to learn those latent correlations. We could exploit this principle in our use case by introducing a simple “cheap” feature, i.e., that is easy to measure and has a more or less obvious correlation to the DS. For instance, we could choose the interval between the image recording date and the date where canopy closure can be observed in the corresponding field experiment. Canopy closure means that neighboring plants touch each other, resulting in a closed field vegetation. In the following, we call this feature DAC. Obviously, this feature has negative values before canopy closure is reached. Alternatively, one could also think about including the days after sowing. The DAC are expected to guide the model to the correct DS by having some correlation with it, e.g., young plants (low DAC) are probably less severely infected, whereas older plants (high DAC) are probably rather severely infected. In addition, the infection probability raises when the plats are in contact. All in all, the MLP neck part is a part of the model, where expert knowledge and known correlations can be integrated. Please note here that in the experiments, we follow another approach to integrate associated knowledge in the model to predict the DS. Nevertheless, the above approach can be a valid, as well.

#### LDL heads.

For each label, the output of the MLP neck is processed by a separate, so called, LDL head. It consists of individual FCL after the MLP neck for each label. The idea behind the individual networks is to enable the model to learn label-individual transformations from the cross-label output of the MLP neck. Thus, the LDL heads are trained to be experts in their label domain and be able to transform the feature space to their regarding label space. When passed through these layers, the features are mixed with a component we call *Feature Mixing*.

#### Feature mixing.

Different labels can contain different amounts of information or be differently difficult to predict. Moreover, they could be (anti-)correlated or complement each other. Thus, we assume that the model profits from a layer that can relate or mix high-level features learned in the previous layers with each other. In a so-called Feature Mixing component, the output layers of all individual LDL Heads are combined linearly. This enables the model to scale and mix information of other labels into the actual labels. The mixing coefficients can be learned during training and are initialized to the unit matrix, i.e., at training start, only the respective label is used. A final FCL for each label maps the mixed features into the corresponding label distribution space. The size of the FCL is determined by the number of discretization or quantization steps that can be different for each label. A softmax activation ensures the outputs of the FCL sum to 1 each. Thus, the FCL approximates the label distribution in the form of a pmf. On this pmf, we can then evaluate our KLD loss functions and, finally, train with the ground truth label distributions.

## Results

In this section, we will introduce our performed experiments. At first, we test if the histogram equalization preprocessing step for the images is really beneficial for the model performance.

**Fig 4:**
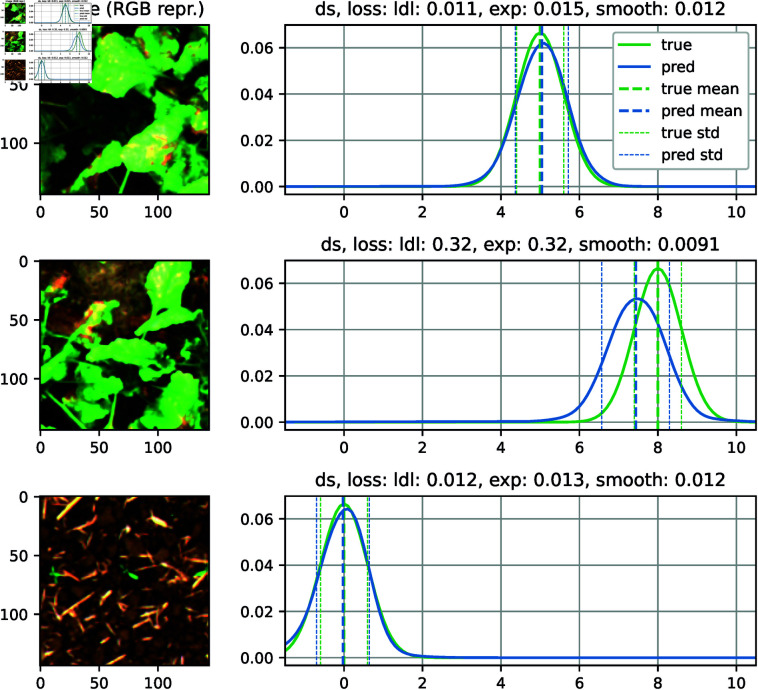
Output of SugarViT. The DS labels are learned as label distributions (green curves). SugarViT outputs again probability distributions (blue curve). The prediction in the end is the expectation value of the output distributions (dashed lines).

In the next experiment, we make use of “cheap” data, i.e., weather data as mentioned in Section *Use case: disease severity estimation*. In our case, we have weather stations in the field measuring basic weather parameters. This data is available for a whole field, thus, many single plant images. With the single images and those cheap labels, we can perform a pretraining of the backbone. However, this is another approach to combine cheap and expensive data than the one mentioned in Section *MLP neck*, the major advantage is that in the final model, only the label of interest is used, which results in a slightly lighter model and decreases inference time. After pretraining, we perform a model training with DS labels, resulting in our SugarViT model. Example predictions of a trained SugarViT model are shown in Fig 4.

In a last experiment, we finally investigate if the pretraining also improves the performance of SugarViT by comparing the finetuned SugarViT with a one only trained by the DS labels. We further compare a non-pretrained SugarViT model to a one that is only trained on with RGB bands information in order to see, whether the beyond-optical spectral information is important.

Before performing the actual experiments, the variances for the DS label distributions must be set, since there is no individual information for each data point, or image. In this work, we model the DS label distributions by normal distributions $N(\cdot |\mu ,\sigma^{2})$ with the experts’ labels as expectation values *μ* and a variance $\sigma^{2}$ that is based on an assumed standard error. We set $\sigma_{DS}=0.6$ as a “human estimation” error. Please note, that this is no empirically found error but rather an educated guess. In the scope of this work, we have only one human estimate per plant and per acquisition date. If multiple experts are involved, one could determine a more realistic error value or even a more realistic pdf. For now, the estimate of a normal distribution with fixed std should be seen as an exemplary choice.

**Fig 5:**
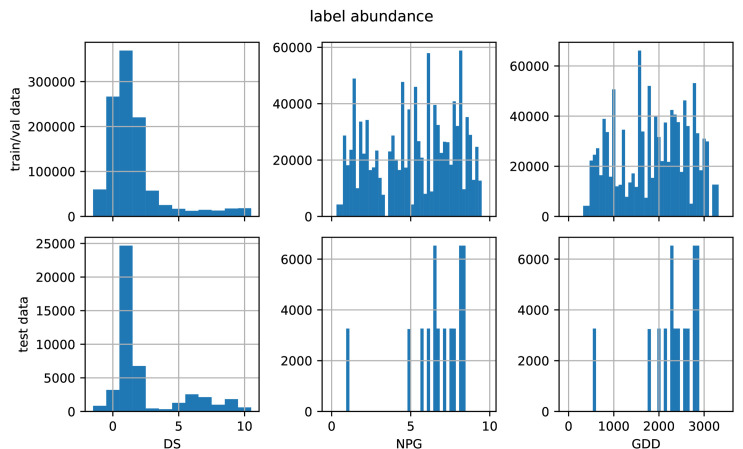
Histograms of available labels for DS, NPG, and GDD separated by train/validation and test data.

In all experiments, we randomly split the training and validation data (cf. Table 1) with initialization seeds assuring reproducibility. For our dataset, the plants have mostly low DS scores in images of the early plant growth, leading to label imbalance, as seen in the histograms in Fig 5. Looking at Table 1, the sizes of the datasets are quite different. In order to minimize the bias and to prevent the model from focusing on few labels and datasets, we use a weighted sampling of the data. The weight of each image is the inverse of the total abundance of its DS label times the size of the respective dataset. Thus, in each training batch, the distribution of datasets and labels is uniformly distributed in average.

Finally, we define a validation metric. Since our model outputs distributions, metrics like root mean squared error or MAE are not appropriate since they do not give information about the overall distribution. Alternatively, we use the mean overlap between the predicted and true label distribution. Since the distributions are pmf, the calculation of the MDO for a batch of *N* instances is


$$\begin{array}{ll} MDO=\frac{1}{N}\overset{N}{\underset{i=1}{\sum }}\underset{y}{\sum }{\left\{\text{min}\left(\mathbb{P}(y|x),\hat{\mathbb{P}}(y|x)\right)\right\}}_{y}. & \; \end{array}$$ (10)

The MDO takes values between 0 and 1 where 1 indicates perfect overlap. For the validation, we use the same weighted sampling as in the training stage, to validate on pseudo-uniform distributed labels. Thus, we respect the prediction quality for each label equally and independent of the total label abundance in the dataset.

### Standardization vs. histogram equalization

Before we perform the training of our SugarViT model, we evaluate how the histogram equalization improves the model performance in favor of a “simpler” standardization preprocessing method. To have a potentially more universal model in the event, we do not assume that we know the dataset as a whole. Thus, we use normalization only based on the information of a single image, as already mentioned in Section *Image normalization*. In the following experiment, we compare the standardization and the histogram equalization method, respectively, with channel-wise and cross-channel calculation. For each method, we perform 10 runs with different initialization seeds with the model configuration given in Table 3. To speed up the training, we only use the arbitrarily chosen datasets Tr01, Tr02, and Tr03 (cf. Table 1) as a subset. Those are randomly split into a training and validation subset by ratio 80*%*:20*%*. This ratio is a good compromise between having a large training set and a sufficiently large validation set. Furthermore, we evaluate the impact of using image augmentation by performing all these experiments with and without using augmentation in the training stage.

**Table 3:** Model configuration for comparison between standardization and histogram equalization.

ViT backbone	
input size	5 × 144*px* × 144*px*
patch size	12*px*
hidden size	512
# hidden layers	4
# attention heads	4
intermediate size	512
activation hidden layers	GELU
dropout hidden layers	0 . 02
dropout attention	0 . 02
**MLP neck**	
layer size	512
layers	3
activation	ReLU
normalization	LayerNorm
dropout	0 . 2
**LDL heads**	
individual MLP layers	2
individual MLP layer size	256
activation	ReLU
dropout	0 . 8
DS quantization steps	131
DS regression limits	[ - 2 . 0, 11 . 0]
DS label distribution std	0 . 6
**Optimizer**	
algorithm	AdamW
initial learning rate	10^-3^
weight decay	0 . 1

**Fig 6:**
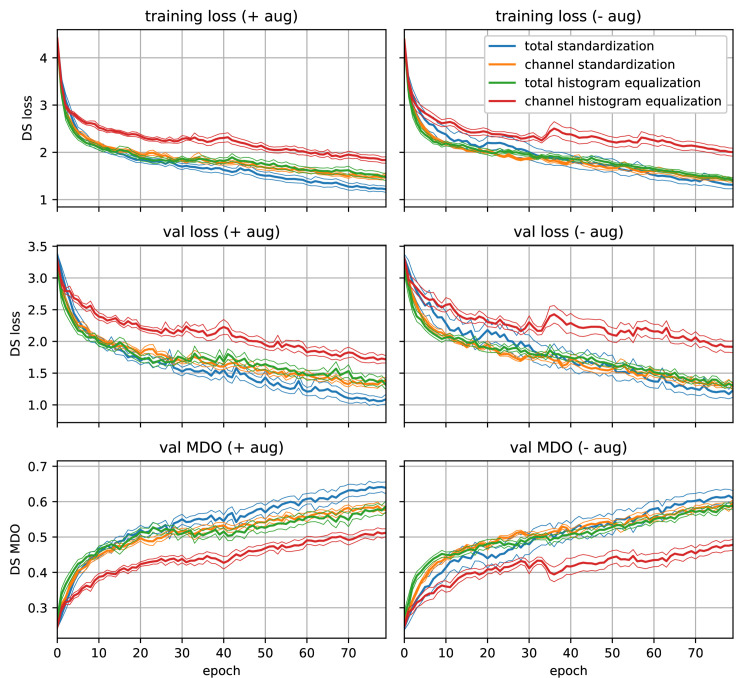
Results of the standardization vs. histogram equalization experiment. For both methods, total and channel-wise variants are shown. For each experiment, 10 runs with different seeds are performed. The bold lines describe the means, whereas the thinner lines are the (positive and negative) standard errors of the respective mean. The left plots show the results with using augmentation during training and the right ones without augmentation. For each variant, training loss (top), validation loss (center), and validation MDO (bottom) are plotted.

The results of a training with 80 epochs are presented in Fig 6. As expected, the total normalization methods perform better than channel-wise normalization. This makes sense, because for DS prediction, an important feature is the difference in radiance of spectral bands, thus, the difference in values across channels. When normalizing the image totally by calculation of cross-channel histograms or mean and std, respectively, this information is preserved, while in the channel-wise normalization it is lost. Nevertheless, channel-wise normalization is more robust against calibration errors of the sensor. Another remarkable observation is, that the standardization method is not only computationally more efficient than histogram equalization, but also performs better. Thus, we find the total (cross-channel) standardization method to be the best performing normalization method, and we will use this method for our SugarViT model. Additionally, the results reveal, that the image augmentation is beneficial for the training process. The effect is low but visible especially for total standardization and channel-wise histogram equalization. Thus, we will use image augmentation in all training stages for further experiments.

### SugarViT pretraining

We perform a pretraining of the SugarViT model on the environmental data labels. This should prepare the model for the plant images by learning low-level features of the plant images. The configuration of our SugarViT model for both pretraining and finetuning stage is listed in Table 4. Please note, that most of the hyperparameters are chosen by educated guesses and are not optimized. A detailed hyperparameter search experiment would have exceeded the scope of this work. Since most of the experiments demonstrated here are of comparisons, the chosen hyperparameters are sufficient to show the model’s capabilities.

**Table 4:** SugarViT model configuration for pretraining and finetuning. For pretraining, the labels NPG and GDD are used. In finetuning stage, the final label of interest, DS, is trained.

ViT backbone	
input size	5 × 144*px* × 144*px*
patch size	12*px*
hidden size	1024
# hidden layers	8
# attention heads	4
intermediate size	1024
activation hidden layers	GELU
dropout hidden layers	0 . 02
dropout attention	0 . 02
**MLP neck**	
layer size	512
layers	3
activation	ReLU
normalization	LayerNorm
dropout	0 . 2
**LDL heads**	
individual MLP layers	2
individual MLP layer size	256
activation	ReLU
normalization	LayerNorm
dropout	0 . 8
NPG quantization steps	100
NPG regression limits	[ - 0 . 5, 11 . 5]
NPG label distribution std	0 . 3
GDD quantization steps	100
GDD regression limits	[ - 5°*C* × d , 3500°*C* × d ]
GDD label distribution std	100°*C* × d
DS quantization steps	131
DS regression limits	[ - 2 . 0, 11 . 0]
DS label distribution std	0 . 6
**Optimizer**	
algorithm	AdamW
initial learning rate	5 × 10^-4^
weight decay	0 . 1
**Learning rate scheduler**	
strategy	cyclic learning rate (linear)
interval	step
maximum lr	1 × 10^-3^
step size (up and down)	500
mode	exponential range
*γ*	0 . 9999

**Fig 7:**
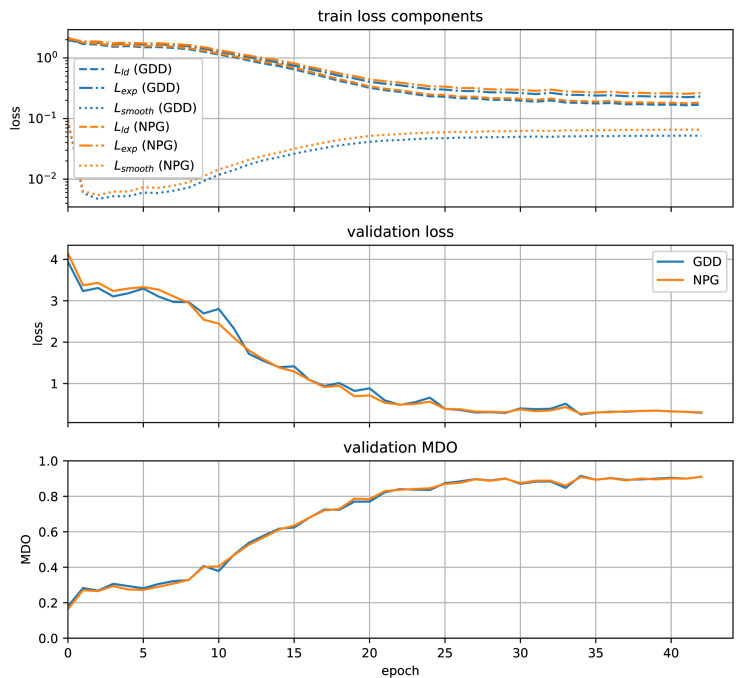
Results of the SugarViT pretraining. The top plot shows training loss by epoch separated into the 3 loss terms (cf. Eq (9)) for GDD (blue) and NPG labels (orange). The two plots below show metrics, namely validation loss (center) and validation MDO (bottom), against the training epoch for both labels.

The results for training and validation loss, as well as the validation MDO are shown in Fig 7. As seen in the training loss component plot, our loss function is indeed invariant under the label scale, as described in Section *Full Kullback-Leibler divergence loss*. Without any scaling parameter, both GDD and NPG labels have a comparable loss, although the scales are very different. We see a convergence of the validation MDO at ca. 90*%* after roughly 40 training epochs for both GDD and NPG labels. As the best model, we take the one with the best validation MDO and use it for the further steps. The results for the training variants with channel dropout and RGB-only information are very similar. Plots can be found in the *??*. If the pretraining was beneficial for the subsequent finetuning regarding convergence speed and prediction quality, is shown in the following section.

### Comparison experiments

In this section, we perform two comparison experiments to justify our choices of model pretraining and using a ViT backbone.

#### Backbone network.

**Fig 8:**
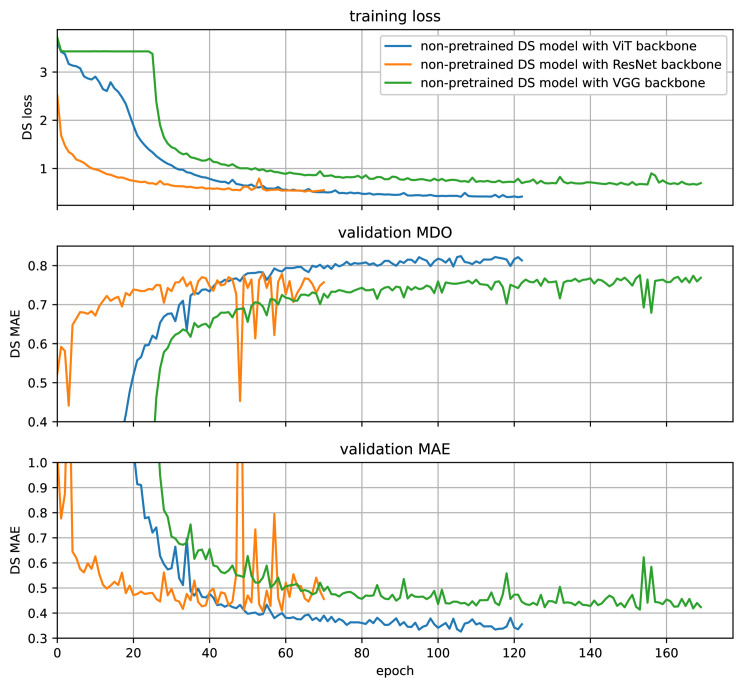
Comparison of backbone networks. Non-pretrained models for prediction of DS are trained using different backbones. The blue lines show our SugarViT model with ViT backbone, whereas the orange lines show the same model but with a ResNet-152 backbone. Green lines show it with a VGG-19 backbone. Training loss (top), validation MDO (center) and validation MAE (bottom), against the training epoch are presented.

First, we take a look at the backbone network that gives SugarViT its name —the ViT. In order to see its benefits, we compare it to another backbone network that has a comparable complexity regarding the number of parameters. Our ViT backbone has about 51.3×10^6^ parameters. As reference backbones, we use a ResNet-152 [38] being similarly complex with 60.2×10^6^ parameters and a VGG-19 [39] with 45.7×10^6^ parameters. To preserve compatibility with our 5-channel input images, we have to change the number of input channels in the first layer. All other model parts are untouched, and we perform a full training from scratch with the same hyperparameters as for SugarViT. As Fig 8 shows, the model with ResNet-152 backbone trains substantially faster. However, when it comes to the validation metrics MDO and MAE, it underperforms our SugarViT regarding the best model metrics. Additionally, the ResNet-152 model seems to be less stable in the metrics due to a volatile behavior beyond roughly 40 epochs. The VGG-19 shows a more stable behavior and a similarly fast training loss convergence. The plateau in the beginning is due to the apparently too high learning rate. Since we perform the same training procedure for all models, the learning rate scheduler is not adapted to the VGG-19 model. Once the learning rate is low enough, the VGG-19 model starts the actual training. However, it underperforms our SugarViT with about the same benchmarks as the ResNet-192 model.

The findings support our statements regarding transformer-based networks demanding more data and patience in training. However, once the data amount is sufficient, they are indeed able to outperform convolution-based networks even with less network complexity like, in our case, with about 15*%* fewer parameters in comparison with ResNet-152.

Nevertheless, the fast training behavior of ResNet-152 and VGG-19 intends that using it for use cases with limited data and resource availability is still a good choice.

#### Pretraining.

**Fig 9:**
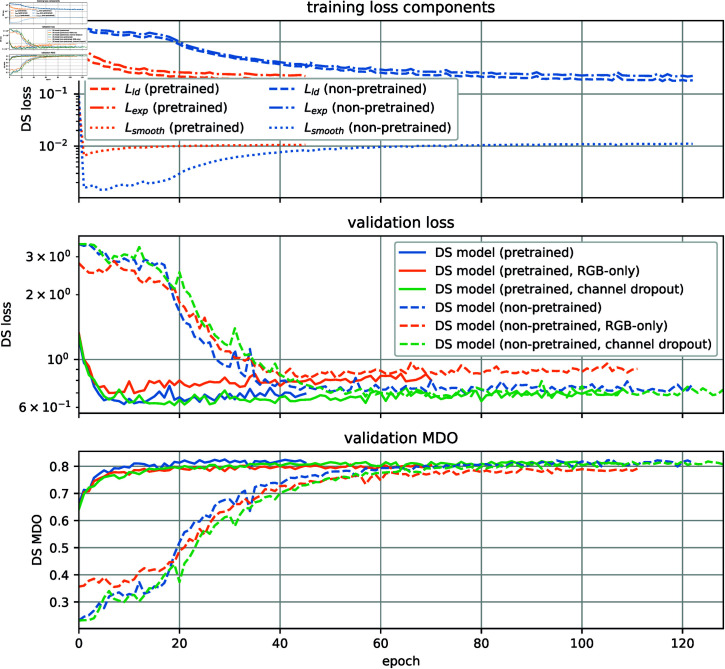
Comparison of the pretrained and non-pretrained SugarViT model. The top plot shows training loss by epoch separated into the 3 loss terms (cf. Eq (9)). Blue lines show the loss components by epoch for the non-pretrained model, orange lines show the ones of the pretrained SugarViT model. The two plots below show metrics, namely validation loss (center) and validation MDO (bottom), against the training epoch. Solid lines show the pretrained, dashed lines the non-pretrained model. The colors denote the three different model variants.

Now, we want to discuss the training and validation metrics of the pretrained SugarViT compared to a non-pretrained model that is trained “from scratch”. Also, we show results for the two training variants mentioned in Section *Results* by using channel dropout or only RGB information during training. For channel dropout, the validation is done without channel dropout, i.e. as for the other methods, in order to preserve comparability. Some performance plots are shown in Fig 9. During the training process, all training loss components converge to similar values, whereby the pretrained models converge, as expected, substantially faster. The validation loss shows similar behavior. In addition, a slight overfitting can be observed for all trainings, as the loss is increasing after a minimum reach at about epochs 10 - 15 for the pretrained and about epochs 50 - 80 for the non-pretrained models. The overfitting can not be observed in terms of validation MDO. For both validation loss and validation MDO the pretrained models reach, besides the faster convergence, slightly better values compared to the non-pretrained models. Overall, the convergence for the channel dropout training is tendentially slower than for the RGB-only and even slower compared to the full model. However, there is no significant benefit visible of using channel dropout during training. Apparently, the model already uses cross-channel information sufficiently well. “Distracting” the model from single-channel traits by canceling out single-channel information seems to be not required.

All in all, we have now determined a stable and well-performing preprocessing method and have assured that choosing a ViT backbone and pretraining the model on environmental data is beneficial for the model performance. The model has the capability to use the multispectral information across channels and is able to learn the low-level features of the plant images. However, we just evaluated the model on the validation dataset so far which is, being a random subset of the training data, quite similar to the training data. In a next step, we want to evaluate our SugarViT models on unseen test data, that is completely unknown by the model in order to see the generalization capabilities of our approach.

### Evaluation on test dataset

Conclusively, we want to evaluate our model on unseen data. Therefore, we use our test dataset Te01 (cf. Table 1). Although we stated that the MAE is not an appropriate metric for the DLDL approach in SugarViT, it can give some insights on the prediction quality in the field usage, since the expectation value of the predicted label distribution is used as the overall model prediction. When analyzing large numbers of plants, the actual predicted label distribution is not as interesting as the actual expected DS value. The purpose of the distribution itself is more of interpretational nature in order to find weaknesses of the model predicting the plant traits of choice. Thus, recap that the MAE of *N* DS predictions is given by


$$\begin{array}{ll} MAE(DS) & =\frac{1}{N}\overset{N}{\underset{i=1}{\sum }}\left|{DS}_{\text{true}}(x_{i})-{DS}_{\text{pred}}(x_{i})\right|\; \end{array}$$ (11)

$$\begin{array}{l} =\frac{1}{N}\overset{N}{\underset{i=1}{\sum }}\left|\text{E}\left[\mathbb{P}(y|x_{i})\right]-\text{E}\left[\hat{\mathbb{P}}(y|x_{i})\right]\right|.\; \end{array}$$ (12)

We evaluated three of our model adaptions, i.e., with using all information, with using channel dropout during training, and with only using RGB channels, each of them with and without using pretraining. Certainly, recognitions can still be incorrect. However, we can apply some techniques to reduce the errors. On the one hand, we can augment the input and use the average of all augmented inputs as the final prediction for the augmented image. One could use any augmentation that we also used during training. However, we just use “simple” augmentations here like mirroring and rotation in order not to reduce the performance, i.e., inference time or usage of computational resources too much. Thus, we can augment one single image to 8 instances in total. Since the model outputs are pmf, we can just add them and renormalize them by dividing by 8. Please note, that using this augmented evaluation is always a tradeoff between prediction robustness and inference time or computational effort. Thus, it is only recommended to use this technique, if enough resources are available in order to guarantee a performant workflow.

**Table 5:** Evaluation metrics for test dataset separated by true DS value. For each training variant, the model with the best validation MDO is chosen. Below the single DS, the metrics for the full dataset are listed, both with and without correcting for the label abundance.

true DS	non-pretrained			pretrained		
	full	ch. dropout	RGB	full	ch. dropout	RGB
-1	**0 . 08**	0 . 12	0 . 10	0 . 11	0 . 11	0 . 09
0	0 . 09	0 . 06	0 . 11	**0 . 04**	0 . 08	0 . 05
1	0 . 23	0 . 23	0 . 27	0 . 23	0 . 23	**0 . 22**
2	0 . 79	**0 . 65**	0 . 77	0 . 74	0 . 76	0 . 70
3	0 . 86	**0 . 84**	1 . 06	0 . 94	0 . 93	1 . 16
4	1 . 28	1 . 23	1 . 06	1 . 34	**1 . 05**	1 . 08
5	0 . 86	0 . 86	0 . 91	1 . 00	**0 . 70**	0 . 75
6	0 . 97	0 . 80	0 . 95	0 . 93	0 . 81	**0 . 72**
7	**0 . 88**	0 . 93	1 . 05	0 . 93	0 . 95	0 . 99
8	**0 . 99**	1 . 02	1 . 22	1 . 00	1 . 12	1 . 35
9	**1 . 10**	1 . 17	1 . 22	1 . 17	1 . 19	1 . 55
10	**0 . 80**	0 . 91	1 . 17	1 . 14	1 . 25	1 . 20
total	0 . 46	**0 . 43**	0 . 50	0 . 46	0 . 45	0 . 46
total (corr.)	0 . 74	**0 . 73**	0 . 82	0 . 80	0 . 76	0 . 82
**true DS**	**non-pretrained**			**pretrained**		
	full	ch. dropout	RGB	full	ch. dropout	RGB
-1	**95 . 53**	93 . 84	94 . 57	94 . 02	94 . 05	94 . 95
0	94 . 03	94 . 51	93 . 22	**95 . 55**	94 . 75	95 . 14
1	**87 . 36**	87 . 14	84 . 86	86 . 26	86 . 49	85 . 70
2	57 . 53	**62 . 52**	57 . 19	59 . 07	58 . 08	61 . 08
3	**60 . 56**	58 . 49	52 . 26	56 . 48	54 . 69	48 . 18
4	44 . 10	45 . 09	**52 . 28**	41 . 59	50 . 73	50 . 16
5	59 . 18	57 . 77	56 . 03	53 . 79	**64 . 96**	60 . 23
6	53 . 53	60 . 84	52 . 67	54 . 63	59 . 27	**61 . 86**
7	**57 . 34**	53 . 78	52 . 76	56 . 93	53 . 47	52 . 12
8	53 . 61	52 . 01	45 . 01	**53 . 69**	48 . 33	40 . 48
9	55 . 28	53 . 11	53 . 41	**55 . 74**	54 . 89	42 . 68
10	**71 . 81**	66 . 66	59 . 36	58 . 24	57 . 35	61 . 52
total	76 . 89	**77 . 53**	74 . 64	76 . 28	76 . 50	75 . 83
total (corr.)	**65 . 74**	65 . 48	62 . 80	63 . 83	64 . 75	62 . 84

As stated in Section *Comparison experiments*, we could observe overfitting in the validation loss, while the MDO is still increasing. We therefore evaluate our training variants by their best models with regard to both metrics separately. It can be observed that the models with the best validation MDO, respectively, perform slightly better in test data evaluation. Apparently, MDO and our KLD based loss have different objectives. Thus, the “better” model is in fact an ambiguous term. Here, we say that the better MAE represents the better model. According to that, the MDO seems to be more sensitive to mean shifts of the distributions, while our loss tends to be more sensitive on variance shifts. Those findings indeed open a discussion of using other training loss metrics like Wasserstein distance but, however, this is beyond the scope of this work. Table 5 shows the MAE and MDO values distributed in each true DS label for those models. The values for the best validation loss models can be found in the *??*. In all evaluations, we use the augmentations mentioned above.

The most remarkable observation is that all models show performance shortcomings for plants with intermediate DS. A reason might be the limited amount of training data in comparison to DS that occur far more frequent. We already try to cope with this imbalance by a weighted sampling as mentioned above. However, the augmented data surely is not “new” data in the actual sense. Unfortunately, those intermediate DS scores are also the most ambiguous ones due to possible bias by margin of (human) interpretation and evaluation. Moreover, we see the importance of the two non-RGB channels since the RGB-only models have a weaker performance in nearly all DS categories. This agrees with the expectation since the symptoms of the CLS disease is mostly visible in the non-optical spectrum, especially in the near-infrared channel. Thus, the NIR and REDGE channel (cf. Table 2) carry valuable information and the experiments show that is in indeed worth using multispectral imaging in favor of ordinary RGB imaging. Another remarkable result is that, apparently, the model channel-dropout does not show significant improvement in terms of generalization to unseen data. It outperforms the fully trained model in only a few DS. In difference to the evaluation on validation data, the non-pretrained models outperform the pretrained ones in most cases for the test dataset. This is quite notable and opens the discussion if the pretraining possibly leads to a slight overfitting to the data and gives another hint about the importance of generalization, especially in agricultural-related use cases. Nevertheless, the idea of pretraining is still important, since training times can be reduced by adaption of one trained backbone to multiple labels of interest. Additionally, in use cases with low data coverage, finetuning existing, pretrained models might be the only way to get performant prediction models.

For the usage of SugarViT in the field, there are still some points to mention regarding further prediction improvements that we want to discuss in the next section.

### Application in the field

Frameworks like [15] help to extract the plant positions and extraction of the single images for large-scale UAV image data. Thereafter, our trained SugarViT model can be applied for new field experiments, enabling fast large-scale DS annotations.

**Fig 10:**
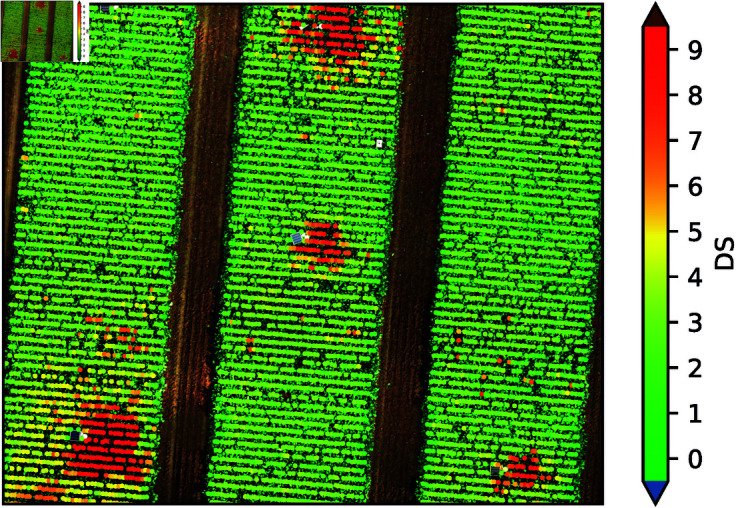
Exemplary application of SugarViT for disease severity prediction on unseen UAV data. Each prediction is completely independent of its surrounding predictions. The model shows a highly consistent prediction behavior.

Fig 10 shows an exemplary application of our model to a test dataset image using the described augmented evaluation. On the other hand, the model does not consider temporal and spatial dependency between the plant images so far. We could further reduce the error rate by correcting single “obvious” outliers that do not fit into the temporal and spatial vicinity of the other plants. Additionally, SugarViT has the advantage to actually output a label distribution. Since we know, with which (fixed) training label standard deviation σ_train_ the model is trained, we can compare the standard deviation of the output (assuming a normal distribution) σ_pred_ with it in order to see, how “confident” the model is in its prediction. Thus, for each output, we can calculate a “confidence”


$$\begin{array}{ll} \tilde{c}=\frac{\sigma_{\text{pred}}}{\sigma_{\text{train}}}\;, & \; \end{array}$$ (13)

that is 1 for an exact conformity of standard deviations. For values $\tilde{c}>1$, the model is more and more unconfident, whereas for values $0<\tilde{c}<1$, the model is over-confident in its decision. However, please note that this is no proper confidence in a statistical sense, since the expectation value still could be completely wrong. Since we cannot know the expectation value in an unlabeled dataset, this purely standard deviation based value is a rather imprecise yet helpful measure of the model’s confidence. Nevertheless, this value can serve as a “warning alert” in a deployed model that it is necessary to cross-check the model output with the estimation of a human expert. The expert could then correct the model output and complicated examples can be identified and used for retraining the model. Thus, the confidence measure and, in the end, LDL enables the possibility for a continuous learning process which is commonly referred to as *Active Learning* with human feedback.

In our model training and evaluation framework, we include some convenience functions to load orthoimages and plant positions as *geopackage* or similar files and evaluate models on. Afterward, the prediction can be exported again to *geopackage* format or, for instance, as *Pandas DataFrame* objects. Thus, we added interfaces to widely used *GIS* software that are frequently used for georeferenced image data.

### Attention maps

**Fig 11:**
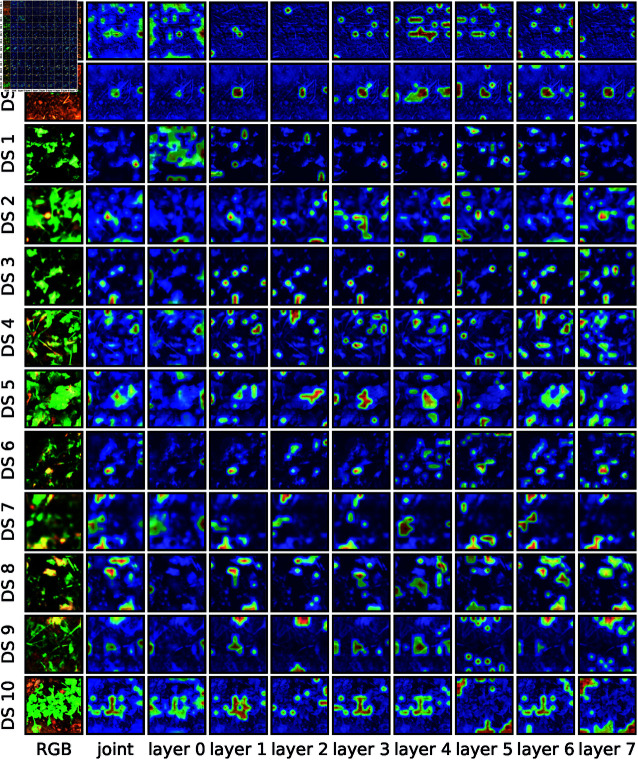
Attention maps for an example image per disease severity class. The first column shows the original input image in its RGB representation. The second column is the joint attention map after performing attention rollout [40]. The following columns are the attention maps of each of the 8 attention layers in our SugarViT model.

Due to the fact that the backbone of our SugarViT model is based on the attention mechanism [11], we can analyze and, ideally, interpret which image feature are more or less important to the model’s decisions. One helpful visualization method for that are so-called *attention maps* [40]. Roughly spoken, attention maps can visualize, “where the model looks at”. The ViT backbone in SugarViT consists of 8 attention layers, with 4 attention heads each, that can in principle be trained to focus on completely different features. In order to accumulate the attention maps of each single layer to one overall map, the technique of *attention rollout* [40] is used. Fig 11 shows the result for one randomly chosen image from the validation dataset per DS class. A main observation is that SugarViT indeed focuses on the plant itself and not, e.g., on the amount of visible soil around it. This is particularly visible for the DS 0 example. Additionally, one observes that the model focuses on multiple image regions that seem to be complementary for the decision-making process, which is exactly the power of the attention mechanism compared to CNN. CNN learn filters that are applied on the whole image. Thus, relations between pixel values can only be covered locally. The attention mechanism allows connecting those local features with other distant images regions and, thus, is considered to have more power of “understanding” the image as a whole.

## Discussion

Our work shows, that simple normalizing methods like standardization can already outperform more sophisticated and expensive normalization methods like histogram equalization (cf. Section *Standardization vs. histogram equalization*). For our use-case where spectral differences carry much information, a total standardization leads to better results than a channel-wise standardization. However, it has to be stated, that the data used in this work has been calibrated by a reflectance panel, so the spectral information is directly comparable. Thus, if the data can not be calibrated by any reason, also the channel-wise standardization may be a good choice, since it also leads to acceptable prediction qualities.

Our comparison experiments have further revealed that, if enough data is given, using a ViT backbone is a good choice. However, for lower data availabilities, also convolution-based backbone networks can reach comparable performances. Furthermore, we have seen that beyond-RGB imagery is beneficial for the prediction of DS in sugar beet plants. This supports the findings of [41] and shows that image information in non-optical bands improve the prediction quality. The ViT backbone is able to use the additional information since the use of channel dropout during training does not show significant improvements in the prediction quality.

Lastly, the pretraining on environmental metadata turns out to be beneficial for the final prediction quality as well as the training speed (cf. Section *SugarViT pretraining*). Accumulating environment-related features like GDD and NPG contain information regarding the plant growth stage and the disease stage, respectively, while being robust against seasonal variations in weather conditions. Thereby, different harvest seasons with very contrasting weather conditions become comparable. Additionally, our pretraining on general, environmental annotation and the subsequent finetuning on the annotation of interest can be an approach for further generalization even on smaller datasets. The pretrained ViT backbone can be seen as a fixed, plant-image-aware feature extractor that learned plant specific traits. On top of that, a smaller model can be trained for different annotation purposes, which accelerates and improves the training procedure substantially. This concept is also widely used in use cases of large language models where the model sizes often exceed computational resources for local training. Pretraining also enables the usage of large-scale image data. Even if, for instance, only few data is labeled with expensive human-expert annotations, the unlabeled majority of the data can still be used in a pretraining stage unfolding the full potential of the collected data. If data labeling originated from multiple human experts that may have ambiguous estimations or assessment guidelines, our usage of LDL is able to incorporate detailed uncertainty information in training process and, finally, in the model.

The use of attention mechanism instead of convolutional networks turns out to make sense in this use case because of the long-distance relations of leaf spots. The interpretability of resulting attention maps on single instances is often questionable. However, they can reveal if the model focuses on the right regions and features itself rather than on spurious correlations.

## Conclusions

Generalization to data from unseen fields with unseen weather conditions and climate is certainly one of the most challenging questions in data-driven machine learning approaches in agriculture. Our findings in Section *Evaluation on test dataset* emphasize that. The data given in the scope of this work comprises already 4 growing seasons but only from some locations in Central Germany. If the model performs comparably well in other regions is at least questionable. Nevertheless, a model that is at least locally accurate already has a high value for increasing the efficiency of DS assessment. Extrapolation to other environmental conditions is challenging, but interpolation on the same field has the potential to save valuable expert working time in the field, where the model can complement a few spot-wise expert annotations on the whole field. As seen in Section *Evaluation on test dataset*, the high data imbalance and label ambiguity still remains challenging, even with our contributions of weighed sampling and LDL, respectively. The disease assessment in individual objects as plants is hard to standardize and schematize by exemplary images, as we, for instance, have in the case of CLS. Therefore, it is important to have a model that incorporates label uncertainties and is transparent in its prediction uncertainties, like in SugarViT.

With this large-scale DS assessment available, further challenges regarding disease assessment can be tackled. The retrieval of DS complemented by further environmental sensors enables, for instance, detailed investigations on disease spread and its modeling. Consequently, our approach could also find application in terms of disease control by, for instance, punctual application of pesticides, lowering costs and environmental impact. Thus, future work regarding this topic will be to use SugarViT for disease spread modeling. Perspectively, the concept behind SugarViT could also be applied in a wide variety of other use cases in the field of UAV-supported phenotyping. [2,42,43]

## Supporting Information

Fig S1 Validation mean distribution overlap (MDO) by training epoch of the SugarViT pretraining for the channel dropout (top) and the RGB-only (bottom) variants. (EPS)

Fig S2 Training loss components by training epoch of the SugarViTDS training for the channel dropout (top) and the RGB-only (bottom) variants. (EPS)

Table S1 Evaluation metrics for test dataset separated by true DSvalue. For each training variant, the model with the best validation loss is chosen. Below the single DSs, the metrics for the full dataset are listed, both with and without correcting for the label abundance.
(EPS)

## Abbreviations:

CLS: Cercospora Leaf Spot
CNN: Convolutional Neural Network
DAC: days after canopy closure
DLDL: Deep Label Distribution Learning
DS: disease severity
FCL: fully-connected layer
GDD: Growing Degree Day
GPT: Generative Pretrained Transformer
HE: Histogram Equalization
KLD: Kullback-Leibler Divergence
LDL: Label Distribution Learning
MAE: mean absolute error
MDO: mean distribution overlap
MLP: Multi-Layer Perceptron
NIR: near-infrared channel
NLP: natural language processing
NPG: number of possible generation
pdf: probability density function
pmf: probability mass function
REDGE: red edge channel
RGB: red, green, and blue channel
std: standard deviation
UAV: Unmanned Aerial Vehicle
ViT: Vision Transformer
